# Promoting health equity in European children: Design and methodology of the prospective EPHE (Epode for the Promotion of Health Equity) evaluation study

**DOI:** 10.1186/1471-2458-14-303

**Published:** 2014-04-02

**Authors:** Krystallia Mantziki, Achilleas Vassilopoulos, Gabriella Radulian, Jean-Michel Borys, Hugues du Plessis, Maria João Gregório, Pedro Graça, Stefaan de Henauw, Svetoslav Handjiev, Tommy LS Visscher, Jacob C Seidell

**Affiliations:** 1 Department of Health Sciences, VU University Amsterdam, De Boelelaan 1085, Amsterdam 1081HV, The Netherlands; 2 Department of Agricultural Economics and Rural Development, Agricultural University of Athens, Athens, Greece; 3 "Carol Davila" University of Medicine and Pharmacy, Bucharest, Romania; 4 EPODE European Network Coordinating Team, Proteines, Paris, France; 5 Faculty of Nutrition and Food Sciences, University of Porto, Porto, Portugal; 6 Directorate General of Health, Lisbon, Portugal; 7 Department of Public Health, Ghent University, Ghent, Belgium; 8 Bulgarian Association for the study of Obesity and related diseases, Sofia, Bulgaria; 9 Research Centre for the Prevention of Overweight, Windesheim University of Applied Sciences Zwolle & VU University, Zwolle, The Netherlands

**Keywords:** Health inequalities, Childhood obesity, EPODE, Dietary intake, Sedentary lifestyle, Sleep

## Abstract

**Background:**

Reducing health inequalities is a top priority of the public health agendas in Europe. The EPHE project aims to analyse the added value of a community-based interventional programme based on EPODE methodology, adapted for the reduction of socio-economic inequalities in childhood obesity. The interventions that will be implemented by this project focus on four energy balance-related behaviours (fruit and vegetable consumption, tap water intake, physical inactivity, sleep duration) and their determinants. This article presents the design of the effect evaluation of the EPHE project.

**Methods/Design:**

This is a prospective two-year follow-up evaluation study, which will collect data on the energy balance-related behaviours and potential environmental determinants of 6–8 year olds, depending on the socio-economic status of the parents. For this purpose a parental self-reported questionnaire is constructed. This assesses the socio-economic status of the parents (5 items) and the dietary (12 items), sedentary (2 items) and sleeping (4 items) behaviour of the child. Alongside potential family-environmental determinants are assessed. The EPHE parental questionnaire will be disseminated in schools of a selected medium-sized city in seven European countries (Belgium, Bulgaria, France, Greece, Portugal, Romania, The Netherlands).

**Discussion:**

This study will evaluate the effects of the EPHE community-based interventional programmes. Furthermore, it will provide evidence for children’s specific energy balance-related behaviours and family environmental determinants related to socio-economic inequalities, in seven European countries.

## Background

Health inequalities between different population groups worldwide and in Europe exist due differences in factors that influence health, such as health related-behaviours, occupational class, education and income [1-3]. Apart from the health impacts of such disparities, the stakes are high even from an economic standpoint. According to the European Parliament, the estimated losses linked to health inequalities had cost around 1.4% of GDP within the European Union in 2011 [4].

Pronounced socio-economic inequalities in non-communicable diseases exist between and within countries in Europe [2,3,5-9], and even at the local level (within-community/neighbourhood) [2,3,5,7,8,10]. Individuals of middle and lower income, occupation class and/or educational level are more likely to develop non-communicable diseases and are more exposed to related risk factors [1-3,5,6,9]. The rates of obesity are higher and increasing more rapidly in those with relatively lower socio-economic status [5-7,9,11,12]. Furthermore, unhealthy dietary habits and less active lifestyle are more common amongst subgroups with a relatively low socio-economic status [8-10,13].

Tackling inequalities in overweight, obesity and related determinants has become a top priority for the European research and policy agendas over the last few years, stressing out the mandatory for action [6,7,9,11,14]. Nevertheless, evidence for the effectiveness of interventions in reducing inequalities in obesity are needed [5,9].

### The EPHE project

Based on the rational above, Epode for the Promotion of Health Equity (EPHE) project was designed. EPHE is a European project running from 2012 to 2015 with the support of the European Commission DG Health and Consumers. Its overall objective is to analyse the added value of community-based approaches based on the EPODE methodology [15,16] in order to reduce inequities associated to childhood obesity and related determinants. In the basis of scientific evidence [10,17-19], four determinants of obesity and overweight will be addressed by the EPHE interventions: promotion of fruit and vegetable intake, tap water intake, active lifestyle and adequate sleep duration. The project involves seven different community-based programmes across Europe (EPODE in France, HEALTHY KIDS in Bulgaria, JOGG in The Netherlands, Maia in Portugal, PAIDEIATROFI in Greece, SETS in Romania, VIASANO in Belgium) and is guided by an EPHE Scientific Advisory Board composed of representatives from 6 European Universities. Based on the results of the baseline measurements, the interventions will focus on the energy balance-related behaviours and their associated environmental determinants where there is the largest gap between high and low socio-economic groups.

The EPHE evaluation study aims (1) to identify the energy balance-related behaviours and explore environmental determinants which are associated with inequalities in childhood obesity and overweight in seven European countries, (2) to assess the effectiveness of EPODE methodology to tackle inequalities in obesity and overweight, (3) to assess the sustainability of potential effects, a year after the termination of the interventions and (4) to provide evidence-based results concerning the inequalities in childhood obesity and overweight across seven European countries. This article aims to describe the design and methodology of the effect evaluation of the EPHE project, which will assess the outcomes of the EPHE selected community-based programmes.

## Methods/Design

The EPHE evaluation plan consists of a prospective two-year follow-up study. It will assess the behavioural change in some energy balance-related behaviours and their associated environmental determinants in children, according to their socio-economic status, and its sustainability over time. The evaluation study will be performed in three measurement periods; baseline (May-June 2013), after the end of the EPHE interventions (May-June 2014) and a year after (May-June 2015). All countries will follow this timeline, with exception of the baseline measurements of France that will be conducted on September 2013, due to practical restrains. The study will include only self-reported measurements by means of a parental questionnaire.

The survey obtained formal declaration from the Medical Ethics Committee of the VU University Medical Centre, that it does not fall under the scope of the Medical Sciences people research Act (WMO). In addition, permission to research in schools was acquired from local community and/or school authorities, where necessary.

### City/Town selection

Each country is represented by a member of its EPHE National Coordination Team, which is a member of the EPHE Operational Board. The National Coordination Team is responsible to guide the Local Project Managers, which are in charge of the data collection in the community level. All the countries will follow a standardised protocol for the selection of the EPHE-city and the data collection, which will be described further in this article.

The evaluation study, as well as the interventions, will be implemented in a medium-sized city/town, where the population shall belong in a wide range of socio-economic statuses. The selected city/town should preferably not have implemented many interventions relevant to nutrition and physical activity addressed to the EPHE target group, in order to prevent of not detecting differences between the socio-economic groups.

To ensure the comparability among the participant communities, the National Coordination Teams must provide a description of the city they will select, before the baseline measurements are conducted. The description will include socio-economic information and health promotion programmes/campaigns conducted in the city/town, along with general information of the selected school(s), including infrastructure.

### Sampling and recruitment

We aim at recruiting at least 150 families with children aged between 6 to 9 years old in every selected city/town with a similar variation regarding age and ethnicity per site, and a preferably low number of different ethnicities (other than the local) per site.

The families will be approached through schools. Every National Coordination Team and Local Team is in charge of committing teachers in the selected schools to enable the distribution and collection of the questionnaires. Teachers, acting as mediators, will approach the families. The National Coordination Teams and Local Project Managers of every country are responsible to engage and guide school directors and teachers in order to recruit the participants. Parents will be provided with an informed consent, describing the purpose of the study.

#### School selection

Of major importance is to account for the variability of the socio-economic status and ethnicity of the sample, both within and between communities. For that reason, the schools should be selected from different neighbourhoods of various socio-economic statuses that assure recruitment of higher and lower socio-economic statuses sample. This should be monitored in the city monitoring at the baseline.

### Socio-economic assessment

Education, social class and income are the most commonly used indicators to assess the socio-economic status in nutritional research [12]. In this study educational level, employment status and income position will be used in order to distinguish the socio-economic status of the parents. Given the current challenging economic instability of European Union, employment status will be assessed instead of the social class. As for some countries it is difficult to evaluate or to obtain quality data, we used the concept of perceived income position, asking parents to self-report their current financial status. Two socio-economic groups will be distinguished based on classification for each indicator: education (low-high), employment status (employed-not employed), income position (good-not good).

### Data collection

In order to ensure the confidentiality of the data, a process to warrant the anonymity will be applied. Each city/town will receive the edited questionnaires labelled with the country’s abbreviation and a three-digit code, indicating the subject’s number. This number will respect to the family name of the subject, indicated in a document that will be kept by the National Coordination Team of every country. As such, only the National Coordination Team will be aware of the subject’s identity, for follow-up purposes. The filled out questionnaires, will be returned sealed up in a provided envelope. The parents will be informed in advance for the process of confidentiality through an information letter, which will include the informed consent as well. Only the children that will return the informed consent indicating agreement of the parent will participate to the study.

The questionnaires will be distributed through schools. More specifically, the teachers will be provided with the labelled questionnaires and envelopes, which will be disseminated by them to the participant children in the class. Following, the children will give them to their parents. The number of distributed questionnaires has to be noted down, in order to monitor the response rates after the collection.

Similarly, after a specified period of one to two weeks, the questionnaires shall be returned back to the teachers. Finally the Local Project Managers will be responsible to collect the returned questionnaires and deliver them to their National Coordination Team. Every National Coordination Team has to keep at least one hard copy of each document, for safety reasons. As mentioned earlier, each local University will have access to their national data.

### Development of questionnaire

A self-reported questionnaire (Additional file 1) is developed, with questions addressed to the parents. The questionnaire will assess information relevant to (1) the family’s socio-economic status and household’s food security level, (2) the child’s energy balance-related behaviours and associated environmental determinants and (3) the parental perception of a healthy body of a child. Based on those measures, it is expected that potential behavioural changes of the child and/or parents will be detected, which will reflect the EPHE- interventions.

The EPHE parental questionnaire was developed using items from relevant, validated questionnaires addressed in European populations. Items derived from validated questionnaires of large European socio-economic surveys [20,21] were chosen to define the socio-economic status. For the assessment of the energy balance-related behaviours and their environmental determinants, items from the ENERGY parent and child questionnaires [22], the Pro-children child questionnaire [23] and its updated version PRO-GREENS [24], were used. These tools have been translated and validated [23,25] in several European languages including some of our interest. Items with intraclass correlation coefficient (ICC) classified as "poor" (ICC < 0.5) were excluded [23,25]. Concerning the household food security level, a short form of the household food security scale developed from the United States Department of Agriculture [26] was used. In order to assess the parent’s perception of their child’s body weight, the pictorial instrument and related questions developed by Collins [27] were used. All items derived from validated questionnaires were adapted for the needs of the EPHE parental questionnaire, where necessary. Additional items were constructed in the cases that no validated items or questionnaires existed to our knowledge.

The questionnaire will be translated in every language, respective to the participant countries and back-translated to English. It is mandatory for all participant countries to use the same version, layout and format of questionnaire.

### Data handling

The questionnaires from all countries will be shipped to the coordinating University in the Netherlands (Vrije University of Amsterdam), where the general analyses will be conducted. A scanned process from the same scanning company will facilitate the data transfer into SPSS files, for all three stages of the evaluation. All the national data will be made available to the national participant-University of country for further analysis.

#### Data cleaning and analysis plan

All the data sets will be checked for missing and double-crossed values. The missing values will be treated by the multiple imputation method, if necessary. The sample will be divided in two groups, according to the socio-economic indicator used in the assessment. Descriptive analysis will include appropriate non-parametric tests for comparing means, in order to detect differences in behaviours and determinants between the two socio-economic groups. The SPSS software 21.0 (IBM Corp., Armonk, NY, USA) will be used for all the analyses.

### Description of selected cities

In each country, EPODE municipality(ies) were selected by the local representatives (National Coordination Team) to participate. Table 1 illustrates the descriptive characteristics of the EPHE cities. All countries are represented by one city, with exceptions to France and Bulgaria where two towns\cities participate.

**Table 1:** Descriptive characteristics of the EPHE cities

**EPHE -city, Country**	**Population *(census)***	**Area**	**Year of entrance in EPODE**
*Mouscron*, Belgium	56.008 *(2011)*	Urban	2006
*Triaditsa,* Bulgaria	65.000 *(2006)*	Urban	2012
*Studentski,* Bulgaria	71.961 *(2006)*	Urban	2012
*Communauté de communes Flandres Lys,* France	34.768 *(2009)*	Rural	1992
*Marousi*, Greece	72.480 *(2011)*	Urban	2010
*Maia,* Portugal	135.306 *(2011)*	Urban	2013
*Otopeni*, Romania	12.671 *(2013)*	Urban	2013
*Zwolle,* The Netherlands	122.625 *(2013)*	Urban	2010

**Figure 1:**
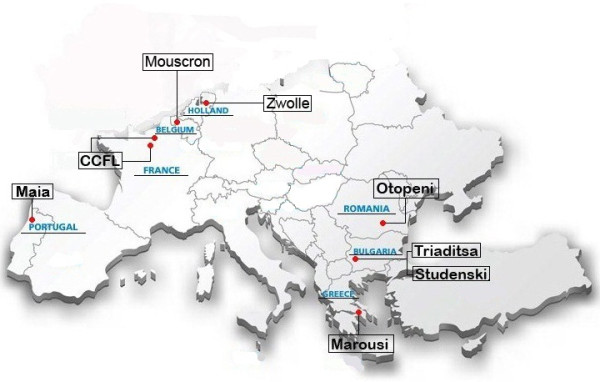
Map of the cities participating in the EPHE programme.

All cities are considered as medium-sized for the country-specific standards. With exception of France, the selected cities are located in urbanised areas. In the cases of Bulgaria, Greece and Portugal, the cities belong to the metropolitan areas of the big cities, in contrary to the rest of the EPHE-cities. Figure 1 illustrates their location. Mouscron (Belgium) is positioned in the west of Belgium, in the French speaking part close to the borders with France. The towns of Triaditsa and Studenski (Bulgaria) belong to the metropolitan area of the capital city Sofia, located in the west of Bulgaria. Communauté de communes Flandres Lys (CCFL) (France) is located in the north-east of France. Marousi town (Greece) is part of the metropolitan of the capital city Athens, positioned in the centre of Greece. Maia city (Portugal) is situated northern to Porto city, in the north of Portugal. Otopeni (Romania) is located in the south of Romania, 15 kilometres northern to the capital, Bucharest. Zwolle (The Netherlands) is positioned in the north part of The Netherlands, 120 kilometres northeast of Amsterdam.

As shown in Table 1, three out of the nine participant municipalities began the implementation of EPODE methodology during the last year, whereas the other six were already committed to an EPODE-like programme. Health campaigns launched by programmes other than EPODE-like, are taking place to the majority of the engaged municipalities. However, these do not always overlap with the target group or the themes of EPHE.

### Description of schools

The sample for the evaluation study will be recruited through schools, selected by the local coordinators of each country. The schools’ selection took into account the need to obtain a mixed sample with children and families from different socioeconomic statuses. In some countries we found these socio-economic variation in the same school, but in other countries, schools belonging to different socioeconomic areas were selected. In Belgium, four schools (three public and one private) from low to medium\high socio-economic areas participate. In Bulgaria ten schools are recruited (nine public and one private) from nine socio-economically mixed areas and one with higher socio-economic status. Greece recruits two public schools, from mixed socio-economic areas, alike to Portugal.

In Romania one public school participates, including students from a broad range of socio-economic statuses. In contrary, in The Netherlands two public schools participate, located in the neighbourhoods from the lowest and the highest socio-economic statuses. Finally, in France two public schools are included, one from a low socio-economic area and the other on from an area with mixed socio-economic status.

### Questionnaire constructs

A total number of 105 items are included in the EPHE parental questionnaire. The average time to fill it out will be approximately 45 minutes.

#### Descriptive and socio-economic variables

Descriptive and socio-economic information are assessed by ten items (Table 2). The descriptive information include age and gender of parent and child. In addition, the size of the household is assessed by two items. For the socio-economic assessment the years of education, labour status and type of working sector of both parents are asked. Alongside, the perception of the household income and its main source are assessed, given ethical restrictions to ask for the exact income. The six-item USDA questionnaire was used to examine the food security level of the household over the past year [26].

**Table 2:** Descriptive and socio-economic variables measured in the EPHE parental questionnaire

**Variable**	**Questionnaire item**	**Response categories**
**Descriptive**		
Questionnaire respondent^a^	This questionnaire is filled in by:	(1) The mother (2) The stepmother (3) The father (4) The stepfather (5) The grandmother (6) The grandfather (7) The caregiver
Age of child	How old is your child?	(1) 6 (2) 7 (3) 8 (4) 9 and above
Age of parent (respondent)^b^	Which age group do you belong to?	(1) 20 and below (2) 20–24 (3) 25–30 (4) 31–35 (5) 36–40 (6) 41 and above
Size of the household^b^	1. How many persons live in your household, including yourself?	1. (1) 2 persons (2) 3–4 persons (3) 5–6 persons (4) More than 6 persons
	2. How many children (below 18 years old) live in your household?	2. (1) 1 child (2) 2 children (3) 3 children (4) a children (5) more than 4 children
**Socio-economic**		
Education^b^	How many years have you/your partner spend in full time study including school?	(1) Less than 6 years (2) 6–8 years (3) 9–11 years (4) 12–14 years (5) 15–17 years (6) More than 17 years (7) I don’t have a spouse/partner
Labour status^b^	How would you define your/your partners’ current labour status?	(1) Carry out a job or profession, including unpaid work for a family business or holding, including an apprenticeship or paid traineeship etc.
		(2) Unemployed (3) Student, further training, unpaid work experience (4) In retirement or early retirement or has given up business (5) Permanently disabled (6) In compulsory military or community service (7) Fulfilling domestic tasks (8) Other inactive person (9) I don’t have a spouse/partner
Sector of employment^c^	Which of the types of organisation you/your spouse work/worked for?	(1) Central or local government (2) Other public sector (such as education and health) (3) A state-owned enterprise (4) A private firm (5) Self-employed (6) Other (7) I don’t have a spouse/partner
Perception of income^c^	Which of the description below comes closest to how you feel about your household’s income nowadays?	(1) Living comfortably on present income (2) Coping on present income (3) Finding it difficult on present income (4) Finding it very difficult on present income
Main source of income^c^	Please consider the income of all household members and any income which may be received by the household as a whole. What is the main source of income in your household?	(1) Wages or salaries (2) Income from self-employment (excluding farming) (3) Income from farming (4) Pensions (5) Unemployment/redundancy benefit (6) Any other social benefits or grants (7) Income from investment, savings, insurance property (8) Income from other sources

^a^Item retrieved from the ENERGY parental questionnaire [22].

^b^Item retrieved or adopted from the European Health Survey questionnaire [21].

^c^Item retrieved from the European Social Survey questionnaire [20].

#### Energy balance related behaviours

Dietary intake and determinants are assessed by sixty-five items, whereas sedentary lifestyle is assessed by fifteen items. Table 3 demonstrates the items of the energy balance-related behaviours of the child (i.e. dietary, sedentary and sleeping behaviours) as indicated in the EPHE parental questionnaire. The consumption of fruits and vegetables is assessed by food frequency questions, referring to a usual week. These items are derived from the Pro children questionnaire [23]*.* A separate item for the potatoes was added in the questionnaire to avoid misleading information, that these are included in the cooked vegetables [23]. Additionally, two items assessing the portions of fruit and vegetables consumed daily are included. The consumption of fruit juices, soft drinks and diet soft drinks is assessed by means of weekly frequency, based on the ENERGY child questionnaire [22,25].

**Table 3:** Dietary, sedentary and sleeping behaviour measured in the EPHE parental questionnaire

**Energy balance-related behaviour**	**Questionnaire item**	**Response categories**
**Dietary Behaviour**		
Fruit consumption^a^	1. How often does your child usually eat fresh fruit?	*8-point scale*; (1) Never (2) Less than 1 day/week (3) 1 day/week (4) 2–4 days a week (5) 5–6 days a week (6) Every day, once/day (7) Every day, twice a day (8) Every day, more than twice/day
Vegetable consumption^a^	1. How often does your child usually eat salad or grated vegetables?	
	2. How often does your child usually eat other raw vegetables?	
	3. How often does your child usually eat cooked vegetables (incl. vegetable soup)?	
Water consumption	1. How many times a day does your child usually drink water?	*6-point scale*; (1) Never (2) Less than once a day (3) Once a day (4) 2–4 times a day (5) 5–6 times a day (6) More than 6 times a day
	2. When your child drinks water, how many glass(es) does (s)he drink?	
Fruit juices consumption^b^	1. How many times a week does your child usually drink fruit juices?	*7-point scale*; (1) Never (2) Less than once a week (3) Once a week (4) 2–4 days a week (5) 5–6 days a week (6) Every day, once a day (7) Every day, more than once a day
	2. On a day that your child drinks fruit juices, how many glass(es), carton(s), bottle(s) or can(s) does (s)he drink?	
Soft drinks consumption^b^	1. How many times a week does your child usually drink soft drinks?	
	2. On a day that your child drinks soft drinks, how many glass(es), can(s) or bottle(s) does (s)he drink?	
Diet soft drinks	1. How many times a week does your child usually drink diet soft drinks?	
	2. On a day that your child drinks diet soft drinks, how many glasses, cans or bottles does (s)he drink?	
**Sedentary behaviour**		*9-point scale*; (1) None at all (2) 30 minutes/day (3) 1.0 hour/day (4) 1.5 hours/day (5) 2.0 hours/day (6) 2,5 hours/day (7) 3.0 hours/day (8) 3.5 hours/day (9) 4.0 or more hours/day
Television viewing^b^	1. About how many hours a day does your child usually watch television in his/her free time?	
Computer time^b^	1. About how many hours a day does your child usually plays computer games or uses the computer for leisure activities?	
**Sleeping behaviour**		
	1. Does your child have a set daily routine for bedtime?^b^	(1) yes (2) no
	2. How many hours a night does your child sleep?^b^	(1) 6–7 hours (2) 8–9 hours (3) 10–11 hours (4) 12 or more hours
	3. What time does your child usually goes to bed?	(1) At 18.00 o’clock (2) At 19.00 o’clock (3) At 20.00 o’clock (4) At 21.00 o’clock (5) At 22.00 o’clock (6) At 23.00 o’clock (7) After 23.00 o’clock
	4. What time does your child usually wake up?	(1) At 05.00 o’clock or earlier (2) At 06.00 o’clock at 07.00 o’clock (3) At 08.00 o’clock (4) At 09.00 o’clock (5) After 09.00 o’clock

^a^Items based on the Pro children child questionnaire [23].

^b^Items derived from the ENERGY parental questionnaire [22].

In order to measure water consumption two frequency questions were constructed, assessing daily intake. Sedentary behaviour is assessed by means of time spent daily in television viewing and time of computer playing, for the week and the weekend days separately. These questions are derived from the ENERGY child questionnaire [22,25]. Furthermore, four questions, partly informed by the ENERGY parent questionnaire and partly constructed, assess the sleeping habits of the child [22]. Finally, three items- one informed by the ENERGY parent questionnaire and the other two by Collins- along with the pictorial instrument created by Collins (1991) [27], assess the parent’s perception of their child’s body weight.

#### Assessment of family environment

The description and questionnaire items of the family environmental variables, mentioned also as determinants of the energy balance-related behaviours, are demonstrated in the Additional file 2. With reference to the Pro Children child questionnaire [23], and its updated version PRO-GREENS [24], and the ENERGY parental questionnaire [22], the family environmental variables can be discriminated into social, physical (i.e. home availability, situation specific habit) and economic (price influence) correlates. Given that the three reference questionnaires make use of slightly different correlates, here they are aggregated into one framework. Therefore, the social correlates include the following mediators for fruit and vegetable consumption: parental demand, parental allowing, active encouragement, facilitating, parental knowledge on recommendations; and the following mediators for fruit juice\soft drink consumption and TV viewing\computer time: paying attention\monitoring, parental allowance, negotiating, communicating health beliefs, avoid negative modelling, parental self-efficacy to manage child’s intake, rewarding\comforting practice. All family environmental variables were assessed by one or two items, using a five response category format. Depending on the item the response categories range a. from (-2) I fully disagree to (2) I fully agree, b. from (1) never to (5) (yes) always, c. from (1) never to (5) every day. Exemptions are the variables assessing the situation of specific habit and the TV availability, where binary response categories are used (i.e. 1.yes, 2. no).

## Discussion

This article describes the methodology of the effect evaluation of the EPHE project, aimed to reduce the socio-economic inequalities in selected energy balance-related behaviours. The EPHE evaluation study is a two-year prospective follow-up survey, which will collect self-reported data of the energy balance-related behaviours of 6–8 year olds and their potential family environmental determinants, depending on the socio-economic level of the parents.

Little research has been conducted to associate childhood obesity and relevant behavioural determinants with socio-economic inequalities in the country level [10]. Nevertheless, obesogenic environments seem to influence more the energy balance-related behaviours of lower socio-economic populations [12,28,29]. Screen exposure of children is inversely associated with parental education [30], whereas lower fruit and vegetable intake is observed more frequently in children with low educated parents [31,32]. However, specific behaviours and determinants of childhood obesity in relation to parental socio-economic status have yet to be identified. The current study will provide evidence for the existence of socio-economic inequalities related to children’s energy balance-related behaviours and potential family environmental determinants, specifically regarding fruit and vegetable consumption, beverage consumption, sedentary lifestyle and sleeping behaviour.

This is one of the few evaluation studies that will assess the effectiveness of interventions in children from lower socio-economic statuses, considering the lack of such evidence [11]. The assessment of potential family environmental correlates, influential to children’s health behaviour, in the socio-economic context is one of the strengths of this study. Furthermore, the use of three different indicators to assess the socio-economic status of the while the most relevant studies use the educational level [30-32], is another strong element. Worth it to mention that these correlates have shown moderate to good reliability and validity in European populations [23,25]. To our knowledge, this is the first study to make use of such correlates in order to evaluate community-based interventions. Next to this, the cross-cultural character of the sample will enable the exploration of inequalities in childhood obesity across different European countries.

However, this study has some limitations. EPODE methodology is conventionally implemented according to the needs and available resources of the community [15,16]. Although this flexibility is an advantage for the implementation of EPODE methodology itself, it complicates the establishment of a robust evaluation framework common for all communities [16]. Considering these, the capacity of the current evaluation study to account for the variations of the local practices and interventions that can influence the effect of the program is limited. Additionally, relative differences (i.e. country-specific) by means of three indicators will approximately determine the socio-economic inequalities within-countries, instead of using more indicators. This reduces the strength of the study to detect absolute inequalities. Self-reported behaviours and determinants may lead to recall bias and eventual socially desirable answers. Furthermore, errors from the constructed items are possible, given that they are not validated. Considering that the family environmental correlates are assessed mostly by one item each, the reliability of the instrument may be violated [22]. Another weakness is the lack of a comparison group, which may result to biased effect size. Finally, this is an observational study and consequently, no conclusions about causality will be drawn.

Considering the strengths and limitations of this evaluation study design, we believe that this study will contribute to the knowledge to explore and describe the health inequalities in sedentary lifestyle, dietary intake and sleep and relevant family environmental across European countries, especially now during the economic crisis in Europe.

## Competing interests

This manuscript arises from the project EPHE – Epode for the Promotion of Health Equity- which has received funding from the European Union, in the framework of the Health Programme, agreement number: 2011 12 09. The study protocol has undergone peer-review from the funding body.

The EPHE Project is funded by the DG SanCO. Additional funding is contributed by Ferrero, Mars, Orangina-Schweppes together with Danone.

The authors declare no competing interests.

## Authors’ contributions

All authors contributed to the design of the study. KM prepared the initial draft of the manuscript. All authors contributed to the writing of the manuscript and approved the final version of the manuscript.

## Pre-publication history

The pre-publication history for this paper can be accessed here:

http://www.biomedcentral.com/1471-2458/14/303/prepub

## Supplementary Material

Additional file 1 Parental questionnaire for the child’s energy balance-related behaviour. Click here for file

Additional file 2 Measurement items of each determinant per energy balance-related behaviour of the EPHE parental questionnaire. Click here for file
