# Supplementary material for: Promoting health equity in European children: Design and methodology of the prospective EPHE (Epode for the Promotion of Health Equity) evaluation study
Source: BMC Public Health. 2014 Apr 2;14:303. doi: 10.1186/1471-2458-14-303 (PMC3997816; doi:10.1186/1471-2458-14-303)
Supplement: Additional file 1 — Parental questionnaire for the child’s energy balance-related behaviour. [file 1471-2458-14-303-S1.pdf]

University Logo

PARENTAL QUESTIONNAIRE FOR THE CHILD'S ENERGY BALANCE-RELATED BEHAVIOR

Final

ephe

EPODE for the Promotion of Health Equity

Student number

PART I

General questions

Socio-demographic information

S1. This questionnaire is filled in by:

- ❑ The mother
- ❑ The stepmother
- ❑ The father
- ❑ The stepfather
- ❑ The grandmother
- ❑ The grandfather
- ❑ The caregiver

S2. How old is your child?

- ❑ 6 years old
- ❑ 7 years old
- ❑ 8 years old
- ❑ 9 years old and above

S3. What is the sex of your child?

- ❑ Boy
- ❑ Girl

S4. Which age group do you belong to?

- ❑ Below 20
- ❑ 20-24
- ❑ 25-30
- ❑ 31-35
- ❑ 36-40
- ❑ above 40

S5. How many years have you/your partner spend in full time study including school?

Me

- ❑ Less than 6 years
- ❑ 6-8 years
- ❑ 9-11 years
- ❑ 12-14 years
- ❑ 15-17 years
- ❑ More than 17 years

Spouse/partner

- ❑ Less than 6 years
- ❑ 6-8 years
- ❑ 9-11 years
- ❑ 12-14 years
- ❑ 15-17 years
- ❑ More than years 17

❑ I don't have a spouse /partner

Student number

S6. How would you define your/your partners' current labour status?

Me

- ❑ Carry out a job or profession, including unpaid work for a family business or holding, including an apprenticeship or paid traineeship etc.
- ❑ Unemployed
- ❑ Student, further training, unpaid work experience
- ❑ In retirement or early retirement or has given up business
- ❑ Permanently disabled
- ❑ In compulsory military or community service
- ❑ Fulfilling domestic tasks
- ❑ Other inactive person

Spouse/partner

- ❑ Carry out a job or profession, including unpaid work for a family business or holding, including an apprenticeship or paid traineeship etc.
- ❑ Unemployed
- ❑ Student, further training, unpaid work experience
- ❑ In retirement or early retirement or has given up business
- ❑ Permanently disabled
- ❑ In compulsory military or community service
- ❑ Fulfilling domestic tasks
- ❑ Other inactive person

- ❑ I don't have a spouse/partner

S7. Which of the types of organisation you/your spouse work/worked for?

Me

- ❑ Central or local government
- ❑ Other public sector (such as education and health)
- ❑ A state-owned enterprise
- ❑ A private firm
- ❑ Self-employed
- ❑ Other

Spouse/partner

- ❑ Central or local government
- ❑ Other public sector (such as education and health)
- ❑ A state-owned enterprise
- ❑ A private firm
- ❑ Self-employed
- ❑ Other

- ❑ I don't have a spouse/partner

Student number

Household information

S8. How many adults live in your household, including yourself?

- ❑ 1 person
- ❑ 2 persons
- ❑ 3-4 persons
- ❑ 5-6 persons
- ❑ More than 6 persons

S9. How many children (below 18 years old) live in your household?

- ❑ 1 child
- ❑ 2 children
- ❑ 3 children
- ❑ 4 children
- ❑ More than 4 children

S10. Which of the description below comes closest to how you feel about your household's income nowadays?

- ❑ Living comfortably on present income
- ❑ Coping on present income
- ❑ Finding it difficult on present income
- ❑ Finding it very difficult on present income

S11. Please consider the income of all household members and any income which may be received by the household as a whole. What is the main source of income in your household?

- ❑ Wages or salaries
- ❑ Income from self-employment (excluding farming)
- ❑ Income from farming
- ❑ Pensions
- ❑ Unemployment/redundancy benefit
- ❑ Any other social benefits or grants
- ❑ Income from investment, savings, insurance property
- ❑ Income from other sources

Student number

For the following statements that people have made about their food situation, please mark how do they apply in your situation, over the last 12 months;

S12. "The food that I bought just didn't last, and I didn't have money to get more". Was that:

- ❑ Often true
- ❑ Sometimes true
- ❑ Never true
- ❑ Don't know

S13. "I couldn't afford to eat balanced meals". Was that:

- ❑ Often true
- ❑ Sometimes true
- ❑ Never true
- ❑ Don't know

S14. In the last 12 months did you in your household ever cut up the size of meals or skip meals because there was not enough money for food?

- ❑ Yes
- ❑ No

If NO, go to question S15

S14a. If yes, how often did this happen?

- ❑ Almost every month
- ❑ Some months but not every month
- ❑ Only 1 or 2 months
- ❑ Don't know

S15. In the last 12 months, did you ever eat less than you felt you should because there wasn't enough money for food?

- ❑ Yes
- ❑ No
- ❑ Don't know

S16. In the last 12 months, were you ever hungry but didn't eat because there wasn't enough money for food?

- ❑ Yes
- ❑ No
- ❑ Don't know

Student number

PART II

a. Some questions about fruit and vegetables

You child's fruit and vegetable consumption habits

F1. How often does your child usually eat fresh fruit?

- ❑ Never
- ❑ Less than one day per week
- ❑ One day per week
- ❑ 2-4 days a week
- ❑ 5-6 days a week
- ❑ Every day, once a day
- ❑ Every day, twice a day
- ❑ Every day, more than twice a day

F2. How often does your child usually eat salad or grated vegetables?

- ❑ Never
- ❑ Less than one day per week
- ❑ One day per week
- ❑ 2-4 days a week
- ❑ 5-6 days a week
- ❑ Every day, once a day
- ❑ Every day, twice a day
- ❑ Every day, more than twice a day

F3. How often does your child usually eat other raw vegetables?

- ❑ Never
- ❑ Less than one day per week
- ❑ One day per week
- ❑ 2-4 days a week
- ❑ 5-6 days a week
- ❑ Every day, once a day
- ❑ Every day, twice a day
- ❑ Every day, more than twice a day

F4. How often does your child usually eat potatoes?

- ❑ Never
- ❑ Less than one day per week
- ❑ One day per week
- ❑ 2-4 days a week
- ❑ 5-6 days a week
- ❑ Every day, once a day
- ❑ Every day, twice a day
- ❑ Every day, more than twice a day

Student number

F5. How often does your child usually eat cooked vegetables (incl. vegetable soup)?

- ❑ Never
- ❑ Less than one day per week
- ❑ One day per week
- ❑ 2-4 days a week
- ❑ 5-6 days a week
- ❑ Every day, once a day
- ❑ Every day, twice a day
- ❑ Every day, more than twice a day

F6. I do not give my child some foods, because they cost too much.

- ❑ I fully agree
- ❑ I agree a somewhat
- ❑ Neither agree nor disagree
- ❑ I disagree somewhat
- ❑ I fully disagree

F7. What do you consider to be the three most important characteristics of your child's meal? *Please tick three boxes*

- ❑ Nutritious
- ❑ Provides energy
- ❑ Exhibits high variety
- ❑ Satisfies my child's liking
- ❑ Home-prepared
- ❑ Organic
- ❑ Vegetarian
- ❑ Taking into account religious requirements

Some questions about fruit only

F8. How many serving portions of fruits does your child eat per day?

- ❑ None
- ❑ 1*(serving portion-country specific)*
- ❑ 2 *(serving portions-country specific)*
- ❑ 3*(serving portions-country specific)*
- ❑ 4 *(serving portions-country specific)*
- ❑ 5 or more *(serving portions-country specific)*

Student number

F9. Do you think that your child eats much or a little fruit?

- ❑ Very much fruit
- ❑ Much fruit
- ❑ Not much, not little
- ❑ Little fruit
- ❑ Very little fruit

F10. How much fruit do you think your child should eat to have a healthy diet?

- ❑ No fruit
- ❑ 1-3 pieces per week
- ❑ 4-6 pieces per week
- ❑ 1 piece per day
- ❑ 2 pieces per day
- ❑ 3 pieces per day
- ❑ 4 pieces per day
- ❑ 5 pieces per day or more

F11. My spouse/partner and/or I often encourage our child to eat fruits.

- ❑ I fully agree
- ❑ I agree somewhat
- ❑ Neither agree nor disagree
- ❑ I disagree somewhat
- ❑ I fully disagree

F12. We often eat fruit together the whole family.

- ❑ I fully agree
- ❑ I agree somewhat
- ❑ Neither agree nor disagree
- ❑ I disagree somewhat
- ❑ I fully disagree

F13. It is a habit of my child to eat fruit every day.

- ❑ I fully agree
- ❑ I agree somewhat
- ❑ Neither agree nor disagree
- ❑ I disagree somewhat
- ❑ I fully disagree

F14. Does your child usually bring fruit with him/her at school?

- ❑ Yes, always
- ❑ Yes, most days
- ❑ Sometimes
- ❑ Seldom
- ❑ Never

Student number

F15. Do you and/or your spouse/partner tell your child to eat fruit every day?

- ❑ Yes, always
- ❑ Yes, most days
- ❑ Sometimes
- ❑ Seldom
- ❑ Never

F16. Is your child allowed to eat as much fruit as (s)he likes at home?

- ❑ Yes, always
- ❑ Yes, most days
- ❑ Sometimes
- ❑ Seldom
- ❑ Never

F17. Are there usually different kinds of fruits available in your home?

- ❑ Yes, always
- ❑ Yes, most days
- ❑ Sometimes
- ❑ Seldom
- ❑ Never

F18. Do you or your spouse/partner usually cut up fruit for your child in between meals?

- ❑ Yes, always
- ❑ Yes, most days
- ❑ Sometimes
- ❑ Seldom
- ❑ Never

Some questions about vegetables only

F19. How many serving portions of vegetables does your child eat per day?

- ❑ None
- ❑ 1*(serving portion-country specific)*
- ❑ 2 *(serving portions-country specific)*
- ❑ 3*(serving portions-country specific)*
- ❑ 4 *(serving portions-country specific)*
- ❑ 5 or more *(serving portions-country specific)*

Student number

F20. Do you think that your child eats many or few vegetables?

- ❑ Very many vegetables
- ❑ Many vegetables
- ❑ Not many, not few
- ❑ Few vegetables
- ❑ Very few vegetables

F21. How many vegetables do you think your child should eat to have a healthy diet?

- ❑ No vegetables
- ❑ 1 - 3 portions (serving spoons) per week
- ❑ 4 - 6 portions (serving spoons) per week
- ❑ 1 portion (serving spoon) every day
- ❑ 2 portions (serving spoons) every day
- ❑ 3 portions (serving-spoons) every day
- ❑ 4 portions (serving-spoons) every day
- ❑ 5 or more portions (serving spoons) every day

F22. My spouse/partner and/or I often encourage our child to eat vegetables every day.

- ❑ I fully agree
- ❑ I agree somewhat
- ❑ Neither agree nor disagree
- ❑ I disagree somewhat
- ❑ I fully disagree

F23. We often eat vegetables together the whole family.

- ❑ I fully agree
- ❑ I agree somewhat
- ❑ Neither agree nor disagree
- ❑ I disagree somewhat
- ❑ I fully disagree

F24. It is a habit for my child to eat vegetables every day.

- ❑ I fully agree
- ❑ I agree somewhat
- ❑ Neither agree nor disagree
- ❑ I disagree somewhat
- ❑ I fully disagree

F25. Does your child usually bring vegetables with him/her at school?

- ❑ Yes, always
- ❑ Yes, most days
- ❑ Sometimes
- ❑ Seldom
- ❑ Never

Student number

F26.Which of the following vegetables does your child like or dislike?

Please, tick one box in every line

	Like very much	Like a bit	Dislike a bit	Dislike very much	Have not tried
Tomatoes	❑	❑	❑	❑	❑
Cucumber	❑	❑	❑	❑	❑
Lettuce	❑	❑	❑	❑	❑
Cabbage	❑	❑	❑	❑	❑
Spinach	❑	❑	❑	❑	❑
Leak	❑	❑	❑	❑	❑
Green beans	❑	❑	❑	❑	❑
Onion	❑	❑	❑	❑	❑
Carrots	❑	❑	❑	❑	❑
Broccoli	❑	❑	❑	❑	❑
Cauliflower	❑	❑	❑	❑	❑
Green peas	❑	❑	❑	❑	❑

F27. Do you and/or your spouse/partner tell your child to eat vegetables every day?

❑ Yes, always
❑ Yes, most days
❑ Sometimes
❑ Seldom
❑ Never

F28. Is your child allowed to eat as many vegetables as (s)he would like at home?

❑ Yes, always
❑ Yes, most days
❑ Sometimes
❑ Seldom
❑ Never

Student number

F29. Are there usually different kinds of vegetables available in your home?

- ❑ Yes, always
- ❑ Yes, most days
- ❑ Sometimes
- ❑ Seldom
- ❑ Never

F30. Do you or your spouse/partner usually cut up vegetables for your child in between meals?

- ❑ Yes, always
- ❑ Yes, most days
- ❑ Sometimes
- ❑ Seldom
- ❑ Never

F31. Are there usually vegetables served with dinner (or lunch) at your home?

- ❑ Yes, always
- ❑ Yes, most days
- ❑ Sometimes
- ❑ Seldom
- ❑ Never

Student number

Some Questions about water

In this section we mean water from the tap or water from bottles (artesian well water, spring water, mineral water and sparkling water).

G1. How many times a day does your child usually drink water?

- ❑ Never
- ❑ Less than once a day
- ❑ Once a day
- ❑ 2-4 times a day
- ❑ 5-6 times a day
- ❑ More than 6 times a day

G2. When your child drinks water, how many glass(es) does (s)he drink?

- ❑ None
- ❑ 1 glass
- ❑ 2 glasses
- ❑ 3 glasses
- ❑ 4 glasses
- ❑ 5 or more glasses

G3. Does your child drink water during mealtime?

- ❑ Always
- ❑ Often
- ❑ Sometimes
- ❑ Not often
- ❑ Never

G4. Does your child drink water between meals?

- ❑ Always
- ❑ Often
- ❑ Sometimes
- ❑ Not often
- ❑ Never

G5. Does your child drink water during/after sports or playing?

- ❑ Always
- ❑ Often
- ❑ Sometimes
- ❑ Not often
- ❑ Never

Student number

Some questions about fruit juices

By fruit juices we mean fruit juices made from concentrated fruit juices and 100% freshly blended fruit juices.

J1. How many times a week does your child usually drink fruit juices?

- ❑ Never
- ❑ Less than once a week
- ❑ Once a week
- ❑ 2-4 days a week
- ❑ 5-6 days a week
- ❑ Every day, once a day
- ❑ Every day, more than once a day

J2. In which situation(s) is your child most likely to drink fruit juices?
You may tick more than one boxes

- ❑ During the weekend
- ❑ Breakfast
- ❑ Lunch
- ❑ Dinner
- ❑ At school
- ❑ While watching television
- ❑ As a thirst quencher between meal
- ❑ During/after sports
- ❑ At birthdays/parties
- ❑ (S)he never drinks fruit juices

J3. On a day that your child drinks fruit juices, how many glass(es), carton(s), bottle(s) or can(s) does (s)he drink? *Please tick a box in every column*

a. Glasses or small cartons (250 ml)

- ❑ None
- ❑ 1 glass/cartons
- ❑ 2 glasses/cartons
- ❑ 3 glasses/cartons
- ❑ 4glasses/cartons
- ❑ 5 or more glasses/cartons

b. Regular cans/cartons/ bottles (330ml)

- ❑ None
- ❑ 1 can/carton/bottle
- ❑ 2 cans/cartons/bottles
- ❑ 3 cans/cartons/ bottles
- ❑ 4 cans/cartons/ bottles
- ❑ 5 or more cans/cartons/ bottles s

Student number

J4. There are fruit juices available at home for my child.

- ❑ Always
- ❑ Often
- ❑ Sometimes
- ❑ Not often
- ❑ Never

J5. I pay attention to the amount of fruit juices my child drinks.

- ❑ Always
- ❑ Often
- ❑ Sometimes
- ❑ Not often
- ❑ Never

J6. If my child asks for fruit juices, I will give it to him/her.

- ❑ Always
- ❑ Often
- ❑ Sometimes
- ❑ Not often
- ❑ Never

J7. My child is allowed to take fruit juices whenever (s)he wants.

- ❑ Always
- ❑ Often
- ❑ Sometimes
- ❑ Not often
- ❑ Never

J8. I negotiate with my child how much fruit juices (s)he is allowed to drink.

- ❑ Always
- ❑ Often
- ❑ Sometimes
- ❑ Not often
- ❑ Never

J9. How often do you tell your child that fruit juices are not good for him/her?

- ❑ Always
- ❑ Often
- ❑ Sometimes
- ❑ Not often
- ❑ Never

J10. How often do you tell your child that fruit juices make him/her fat?

- ❑ Always
- ❑ Often
- ❑ Sometimes
- ❑ Not often
- ❑ Never

J11. If I would like to drink fruit juices, I would restrain myself because of the presence of my child.

- ❑ Always
- ❑ Often
- ❑ Sometimes
- ❑ Not often
- ❑ Never

Student number

J12. If I prohibit my child from drinking fruit juices, (s)he tries to drink anyway.

- ❑ Always
- ❑ Often
- ❑ Sometimes
- ❑ Not often
- ❑ Never

J13. If I prohibit my child from drinking fruit juices, I find it difficult to stick to my rule(s) if (s)he starts negotiating.

- ❑ Always
- ❑ Often
- ❑ Sometimes
- ❑ Not often
- ❑ Never

J14. I give fruit juices to my child as a reward or to comfort him/her.

- ❑ Always
- ❑ Often
- ❑ Sometimes
- ❑ Not often
- ❑ Never

J15. How often do you and/or your spouse/partner drink fruit juices together with your child?

- ❑ Never
- ❑ Less than once a week
- ❑ Once a week
- ❑ 2-4 days a week
- ❑ 5-6 days a week
- ❑ Every day, once a day
- ❑ Every day, more than once a day

Student number

Some questions about soft drinks

By soft drinks, we mean fizzy drinks and fruit squash, but NOT diet drinks and fruit juices. Examples:

Fizzy drinks are: cola, 7-up, Pepsi, Fanta, Sprite etc.

Fruit squash/ cordials: Ice tea, lemonade etc.

Sport and energy drinks: lucozade, redbull etc.

K1. How many times a week does your child usually drink soft drinks?

- ❑ Never
- ❑ Less than once a week
- ❑ Once a week
- ❑ 2-4 days a week
- ❑ 5-6 days a week
- ❑ Every day, once a day
- ❑ Every day, more than once a day

K2. In which situation(s) is your child most likely to drink soft drinks?

You may tick more than one boxes

- ❑ During the weekend
- ❑ Breakfast
- ❑ Lunch
- ❑ Dinner
- ❑ At school
- ❑ While watching television
- ❑ As a thirst quencher between meal
- ❑ During/after sports
- ❑ At birthdays/parties
- ❑ (S)he never drinks fruit juices

Student number

K3. On a day that your child drinks soft drinks, how many glass(es), can(s) or bottle(s) does (s)he drink? *Please tick a box in every column*

a. Glasses or small bottles (250 ml)

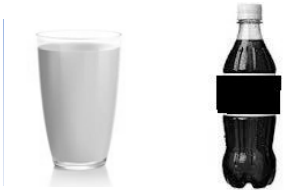

- ☐ None
- ☐ 1 glass/small bottle
- ☐ 2 glasses/small bottles
- ☐ 3 glasses/ small bottles
- ☐ 4glasses/ small bottles
- ☐ 5 or more glasses/ small bottles

b. Cans (330ml)

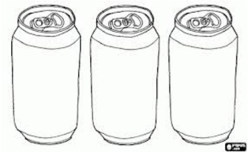

- ☐ None
- ☐ 1 can
- ☐ 2 cans
- ☐ 3 cans
- ☐ 4 cans
- ☐ 5 or cans

c. Bottles (500 ml)

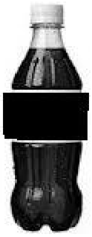

- ☐ None
- ☐ 1 bottle
- ☐ 2 bottles
- ☐ 3 bottles
- ☐ 4 bottles
- ☐ 5 or more bottles

K4. There are soft drinks available at home for my child.

- ☐ Always
- ☐ Often
- ☐ Sometimes
- ☐ Not often
- ☐ Never

K5. I pay attention to the amount of soft drinks my child drinks.

- ☐ Always
- ☐ Often
- ☐ Sometimes
- ☐ Not often
- ☐ Never

K6. If my child asks for soft drinks, I will give it to him/her.

- ☐ Always
- ☐ Often
- ☐ Sometimes
- ☐ Not often
- ☐ Never

K7. My child is allowed to take soft drinks whenever (s)he wants.

- ☐ Always
- ☐ Often
- ☐ Sometimes
- ☐ Not often
- ☐ Never

Student number

K8. How often do you tell your child that soft drinks are not good for him/her?

- ❑ Always
- ❑ Often
- ❑ Sometimes
- ❑ Not often
- ❑ Never

K9. How often do you tell your child that soft drinks make him/her fat?

- ❑ Always
- ❑ Often
- ❑ Sometimes
- ❑ Not often
- ❑ Never

K10. If I would like to drink soft drinks, I would restrain myself because of the presence of my child.

- ❑ Always
- ❑ Often
- ❑ Sometimes
- ❑ Not often
- ❑ Never

K11. If I prohibit my child from drinking soft drinks, (s)he tries to drink anyway.

- ❑ Always
- ❑ Often
- ❑ Sometimes
- ❑ Not often
- ❑ Never

K12. If I prohibit my child from drinking soft drinks, I find it difficult to stick to my rule(s) if (s)he starts negotiating

- ❑ Always
- ❑ Often
- ❑ Sometimes
- ❑ Not often
- ❑ Never

K13. I give soft drinks to my child as a reward or to comfort him/her.

- ❑ Always
- ❑ Often
- ❑ Sometimes
- ❑ Not often
- ❑ Never

Student number

K14. How often do you (one parent/ spouse/partner or both) drink soft drinks together with your child?

- ☐ Never
- ☐ Less than once a week
- ☐ Once a week
- ☐ 2-4 days a week
- ☐ 5-6 days a week
- ☐ Every day, once a day
- ☐ Every day, more than once a day

Student number

Some questions about <u>diet</u> soft drinks

D1. How many times a week does your child usually drink diet soft drinks?

- ☐ Never
- ☐ Less than once a week
- ☐ Once a week
- ☐ 2-4 days a week
- ☐ 5-6 days a week
- ☐ Every day, once a day
- ☐ Every day, more than once a day

D2. On a day that your child drinks diet soft drinks, how many glasses, cans or bottles does (s)he drink? *Please tick a box in every column*

a. Glasses or small bottles (250 ml)

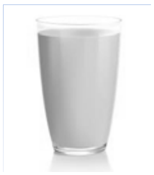

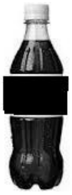

- ☐ None
- ☐ 1 glass/small bottle
- ☐ 2 glasses/small bottles
- ☐ 3 glasses/ small bottles
- ☐ 4 glasses/ small bottles
- ☐ 5 or more glasses/ small bottles

b. Cans (330ml)

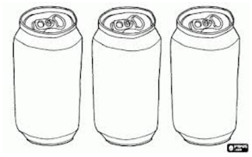

- ☐ None
- ☐ 1 can
- ☐ 2 cans
- ☐ 3 cans
- ☐ 4 cans
- ☐ 5 or cans

c. Bottles (500 ml)

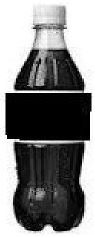

- ☐ None
- ☐ 1 bottle
- ☐ 2 bottles
- ☐ 3 bottles
- ☐ 4 bottles
- ☐ 5 or more bottles

Student number

Part III

Questions about your child's screen behaviour

For the following questions, when we say watching television, we mean also watching DVD's, videos and watching films in the computer.

By computer games, we mean also games in mobile phone/computer/ tablets and consoles (e.g. playstation)

T1. About how many hours a day does your child usually watch television in his/her free time?

a. Weekdays (average of all weeks)

- ☐ None at all
- ☐ 30 minutes/day
- ☐ 1,0 hour/day
- ☐ 1,5 hours/day
- ☐ 2,0 hours/ day
- ☐ 2,5 hours/ day
- ☐ 3,0 hours/ day
- ☐ 3,5 hours/ day
- ☐ 4,0 or more hours/ day

b. Weekend days (average of all weekends)

- ☐ None at all
- ☐ 30 minutes/day
- ☐ 1,0 hour/day
- ☐ 1,5 hours/day
- ☐ 2,0 hours/ day
- ☐ 2,5 hours/ day
- ☐ 3,0 hours/ day
- ☐ 3,5 hours/ day
- ☐ 4,0 or more hours/ day

T2. TV is available in my child's room.

- ☐ Yes
- ☐ No

T3. How often is the TV on during dinner (supper/ evening meal) in your home?

- ☐ Every day
- ☐ 4-6 days a week
- ☐ 1-3 days a week
- ☐ Less than 1 day week
- ☐ Never

Student number

T4. About how many hours a day does your child usually plays computer games or uses the computer for leisure activities? *Please tick one box in every column*

a. Weekdays (average of all weeks)

- ❑ None at all
- ❑ 30 minutes/day
- ❑ 1,0 hour/day
- ❑ 1,5 hours/day
- ❑ 2,0 hours/ day
- ❑ 2,5 hours/ day
- ❑ 3,0 hours/ day
- ❑ 3,5 hours/ day
- ❑ 4,0 or more hours/ day

b. Weekend days (average of all weekends)

- ❑ None at all
- ❑ 30 minutes/day
- ❑ 1,0 hour/day
- ❑ 1,5 hours/day
- ❑ 2,0 hours/ day
- ❑ 2,5 hours/ day
- ❑ 3,0 hours/ day
- ❑ 3,5 hours/ day
- ❑ 4,0 or more hours/ day

For questions T5-T13, please tick one box in column a AND b.

T5. I pay attention to the amount of the time my child:

a. watches TV

- ❑ Always
- ❑ Often
- ❑ Sometimes
- ❑ Not often
- ❑ Never

b. plays computer games

- ❑ Always
- ❑ Often
- ❑ Sometimes
- ❑ Not often
- ❑ Never

T6. If my child asks if (s)he is allowed to watch TV/play computer games I will allow it:

a. watch TV

- ❑ Always
- ❑ Often
- ❑ Sometimes
- ❑ Not often
- ❑ Never

b. play computer games

- ❑ Always
- ❑ Often
- ❑ Sometimes
- ❑ Not often
- ❑ Never

Student number

T7. My child is allowed to watch TV/play computer games whenever (s)he wants:

a. watch TV

- ❑ Always
- ❑ Often
- ❑ Sometimes
- ❑ Not often
- ❑ Never

b. play computer games

- ❑ Always
- ❑ Often
- ❑ Sometimes
- ❑ Not often
- ❑ Never

T8. I negotiate with my child how much (s)he is allowed to watch TV/play computer games:

a. watch TV

- ❑ Always
- ❑ Often
- ❑ Sometimes
- ❑ Not often
- ❑ Never

b. play computer games

- ❑ Always
- ❑ Often
- ❑ Sometimes
- ❑ Not often
- ❑ Never

T9. If I would like to watch TV/use the computer for leisure time, I would restrain myself because of the presence of my child.

a. watch TV

- ❑ Always
- ❑ Often
- ❑ Sometimes
- ❑ Not often
- ❑ Never

b. use computer

- ❑ Always
- ❑ Often
- ❑ Sometimes
- ❑ Not often
- ❑ Never

Student number

T10. If I prohibit my child to watch TV/use the computer for leisure time, (s)he would do it anyway.

a. watch TV

- ❑ Always
- ❑ Often
- ❑ Sometimes
- ❑ Not often
- ❑ Never

b. use computer

- ❑ Always
- ❑ Often
- ❑ Sometimes
- ❑ Not often
- ❑ Never

T11. If I prohibit my child from watching TV/playing computer games, I find it difficult to stick to my rule(s) if (s)he starts negotiating:

a. watching TV

- ❑ Always
- ❑ Often
- ❑ Sometimes
- ❑ Not often
- ❑ Never

b. playing computer games

- ❑ Always
- ❑ Often
- ❑ Sometimes
- ❑ Not often
- ❑ Never

T12. I allow my child to watch TV/ play computer games as a reward or to comfort him/her.

a. watch TV

- ❑ Always
- ❑ Often
- ❑ Sometimes
- ❑ Not often
- ❑ Never

b. play computer games

- ❑ Always
- ❑ Often
- ❑ Sometimes
- ❑ Not often
- ❑ Never

Student number

T13. How often do you and/or spouse/ partner watch television/play computer games together with your child?

a. watch TV

- ❑ Always
- ❑ Often
- ❑ Sometimes
- ❑ Not often
- ❑ Never

b. play computer games

- ❑ Always
- ❑ Often
- ❑ Sometimes
- ❑ Not often
- ❑ Never

T14. How often do you tell to your child that watching TV/playing computer games is not good for him/her?

- ❑ Always
- ❑ Often
- ❑ Sometimes
- ❑ Not often
- ❑ Never

T15. How often do you tell to your child that watching TV/playing computer games makes him/her fat?

- ❑ Always
- ❑ Often
- ❑ Sometimes
- ❑ Not often
- ❑ Never

a. Some questions about your child's sleep

L1. Does your child have a set daily routine for bedtime?

- ❑ Yes
- ❑ No

Student number

L2. How many hours a night does your child sleep?

Please tick one box in every column

a. Weekdays(average per night)

- ❑ 6-7 hours
- ❑ 8-9 hours
- ❑ 10-11 hours
- ❑ 12 or more hours

b. Weekend days (average per night)

- ❑ 6-7 hours
- ❑ 8-9 hours
- ❑ 10-11 hours
- ❑ 12 or more hours

L3. What time does your child usually goes to bed?

a. Weekdays (average of all weeks)

- ❑ At 18.00 o'clock
- ❑ At 19.00 o'clock
- ❑ At 20.00 o'clock
- ❑ At 21.00 o'clock
- ❑ At 22.00 o'clock
- ❑ At 23.00 o'clock
- ❑ After 23.00 o'clock

b. Weekend days (average of all weekends)

- ❑ At 18.00 o'clock
- ❑ At 19.00 o'clock
- ❑ At 20.00 o'clock
- ❑ At 21.00 o'clock
- ❑ At 22.00 o'clock
- ❑ At 23.00 o'clock
- ❑ After 23.00 o'clock

L4.What time does your child usually wake up?

a. Weekdays(average of all weeks)

- ❑ At 05.00 o'clock or earlier
- ❑ At 06.00 o'clock
- ❑ At 07.00 o'clock
- ❑ At 08.00 o'clock
- ❑ At 09.00 o'clock
- ❑ After 09.00 o'clock

b. Weekend days (average of all weekends)

- ❑ At 05.00 o'clock or earlier
- ❑ At 06.00 o'clock
- ❑ At 07.00 o'clock
- ❑ At 08.00 o'clock
- ❑ At 09.00 o'clock
- ❑ After 09.00 o'clock

Student number

c. Some questions about your child's weight

W1. What do you think about your child's weight?

- ❑ My child's weight is ok
- ❑ My child's weight is a bit too much
- ❑ My child's weight is way too much
- ❑ My child's weight is a bit too little
- ❑ My child's weight is way too little

Please look carefully the picture below and answer the following questions:

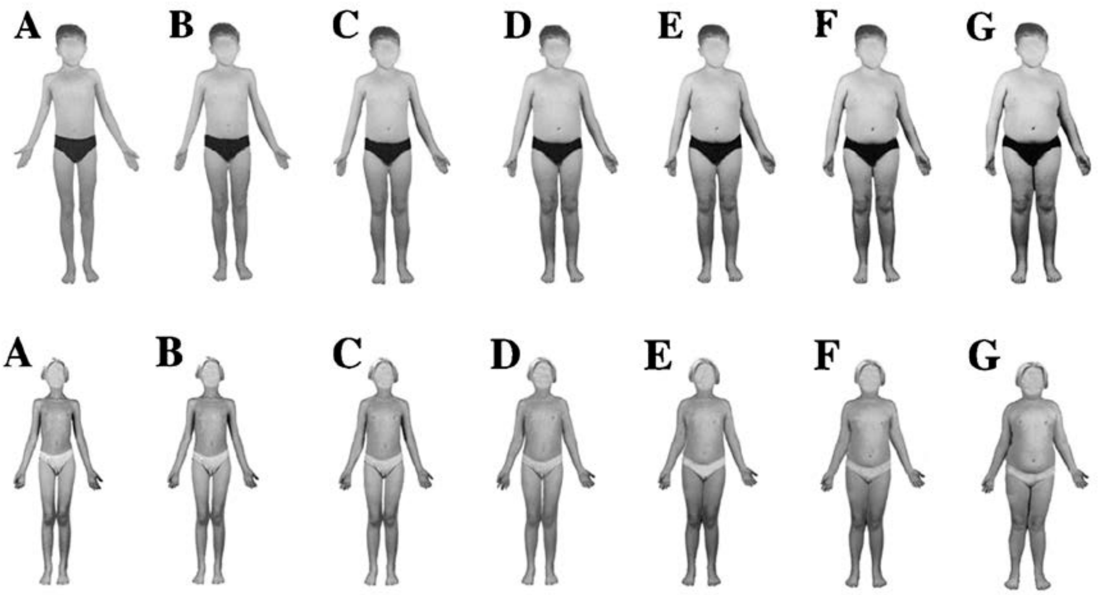

W2. Choose at the figure that you think most accurately represents your child at this moment

Write down the letter of the figure

☐

W3. Choose the figure that you think that represents the one you think would be the best for your child

Write down the letter of the figure

☐

Thank you for completing this questionnaire!
