# Supplementary material for: Promoting health equity in European children: Design and methodology of the prospective EPHE (Epode for the Promotion of Health Equity) evaluation study
Source: BMC Public Health. 2014 Apr 2;14:303. doi: 10.1186/1471-2458-14-303 (PMC3997816; doi:10.1186/1471-2458-14-303)
Supplement: Additional file 2 — Measurement items of each determinant per energy balance-related behaviour of the EPHE parental questionnaire. [file 1471-2458-14-303-S2.pdf]

Additional file 2. Measurement items of each determinant per energy balance-related behaviour of the EPHE parental questionnaire.

	Family Environmental categorised correlates							
	Description	Fruit [a]	Vegetables [a]	Water	Fruit juices [b]	Soft drinks [b]	Television viewing [b]	Computer time
Social Environment								
Parenting practices	Assessing parenting rules and practices applied to the child in relation to Energy balance-related behaviours	1. My spouse/partner and/or I often encourage our child to eat fruits. 2. Do you and/or your spouse/partner tell your child to eat fruit every day? 3. Is your child allowed to eat as much fruit as (s)he likes at home? 4. Do you or your spouse/partner usually cut up fruit for your child in between meals?	1. My spouse/partner and/or I often encourage our child to eat vegetables every day. 2. Do you and/or your spouse/partner tell your child to eat vegetables every day? 3. Is your child allowed to eat as many vegetables as (s)he would like at home? 4. Do you or your spouse/partner usually cut up vegetables for your child in between meals? 5. Are there usually vegetables served with dinner (or lunch) at your home?		1. I pay attention to the amount of fruit juices my child drinks. 2. If my child asks for fruit juices, I will give it to him/her. 3. My child is allowed to take fruit juices whenever (s)he wants. 4. I negotiate with my child how much fruit juices (s)he is allowed to drink. 5. How often do you tell your child that fruit juices are not good for him/her? 6. How often do you tell your child that fruit juices make him/her fat? 7. If I would like to drink fruit juices, I would restrain myself because of the presence of my child.	1. I pay attention to the amount of soft drinks my child drinks. 2. If my child asks for soft drinks, I will give it to him/her. 3. My child is allowed to take soft drinks whenever (s)he wants. 3. How often do you tell your child that soft drinks are not good for him/her? 4. How often do you tell your child that soft drinks make him/her fat? 5. If I would like to drink soft drinks, I would restrain myself because of the presence of my child. 6. If I prohibit my child from drinking soft drinks, I find it difficult to stick to	1. I pay attention to the amount of the time my child watches TV. 2. If my child asks if (s)he is allowed to watch TV I will allow it. 3. My child is allowed to watch TV whenever (s)he wants. 4. I negotiate with my child how much (s)he is allowed to watch TV. 5. If I would like to watch TV for leisure time, I would restrain myself because of the presence of my child. 6. If I prohibit my child from watching TV, I find it difficult to stick to my rule(s) if (s)he starts negotiating. 7. I allow my child to watch TV as a	1. I pay attention to the amount of the time my child plays computer games. 2. If my child asks if (s)he is allowed to play computer games I will allow it. 3. My child is allowed to play computer games whenever (s)he wants. 4. I negotiate with my child how much (s)he is allowed to play computer games. 5. If I would like to use the computer for leisure time, I would restrain myself because of the presence of my child. 6. If I prohibit my child from playing computer games, I find it difficult to stick to my rule(s)

Additional file 2. Measurement items of each determinant per energy balance-related behaviour of the EPHE parental questionnaire *(continued).*

					8. If I prohibit my child from drinking fruit juices, I find it difficult to stick to my rule(s) if (s)he starts negotiating.<br>9. I give fruit juices to my child as a reward or to comfort him/her.	my rule(s) if (s)he starts negotiating.<br>7. I give soft drinks to my child as a reward or to comfort him/her.	reward or to comfort him/her.<br>8. How often do you tell to your child that watching TV/playing computer games is not good for him/her?<br>9. How often do you tell to your child that watching TV/playing computer games makes him/her fat?	if (s)he starts negotiating.<br>7. I allow my child to play computer games as a reward or to comfort him/her.<br>8. How often do you tell to your child that watching TV/playing computer games is not good for him/her?<br>9. How often do you tell to your child that watching TV/playing computer games makes him/her fat?
Parental knowledge	Assessing parental perception of child's dietary intake and knowledge of recommended dietary intake	1. Do you think that your child eats much or a little fruit?<br>2. How much fruit do you think your child should eat to have a healthy diet?	1. Do you think that your child eats many or few vegetables?<br>2. How many vegetables do you think your child should eat to have a healthy diet?					
Performing EBRB together	Assessing the frequency that parents conduct the EBRB together with their child	1. We often eat fruit together the whole family.	1. We often eat vegetables together the whole family.		1. How often do you and/or your spouse/partner drink fruit juices together with your child?	1. How often do you (one parent/ spouse/partner or both) drink soft drinks together with your child?	1. How often do you and/or spouse/ partner watch television together with your child?	1. How often do you and/or spouse/ partner play computer games together with your child?
Child's nagging behaviour	Assess the child's reaction when parent prohibits an				1. If I prohibit my child from	1. If I prohibit my child from	1. If I prohibit my child to watch TV	1. If I prohibit my child to use the

Additional file 2. Measurement items of each determinant per energy balance-related behaviour of the EPHE parental questionnaire *(continued).*

	action				drinking fruit juices, (s)he tries to drink anyway.	drinking soft drinks, (s)he tries to drink anyway.	for leisure time, (s)he would do it anyway.	computer for leisure time, (s)he would do it anyway.
Physical Environment								
Home availability	Assessing whether specific food products or equipment related to Energy balance-related behaviours are available at home for the child	1. Are there usually different kinds of fruits available in your home?	1. Are there usually different kinds of vegetables available in your home?		1. There are fruit juices available at home for my child.	1. There are soft drinks available at home for my child.	1. TV is available in my child's bedroom.	
Situation specific habit	Assessing the specific situations that child habitually conducts an EBRB	1. It is a habit of my child to eat fruit every day.<br>2. Does your child usually bring fruit with him/her at school?	1. It is a habit for my child to eat vegetables every day.<br>2. Does your child usually bring vegetables with him/her at school?	1. Does your child drink water during mealtime?<br>2. Does your child drink water between meals?<br>3. Does your child drink water during/after sports or playing?	1. In which situation(s) is your child most likely to drink fruit juices?	1. In which situation(s) is your child most likely to drink soft drinks?	1. How often is the TV on during dinner (supper/ evening meal) in your home?	
				**Family Environmental non-categorised correlates**				
Economic Environment								
Price influence	Assessing the influence of price on parent's food choices for their child	1. I do not give my child some foods because they cost too much [b]						
Food characteristics	Assessing characteristics that influence parents' food choice for the child's meal	1. What do you consider to be the most important characteristic (s) of your child's meal? [b]						

[a] Items of these categories are based on the Pro children [23] and PRO-GREENS [24] child questionnaires.

[b] Items of these categories are derived from the ENERGY parental questionnaire [22].
